# The protein tyrosine phosphatase 1B inhibitor MSI-1436 stimulates regeneration of heart and multiple other tissues

**DOI:** 10.1038/s41536-017-0008-1

**Published:** 2017-03-03

**Authors:** Ashley M. Smith, Katie K. Maguire-Nguyen, Thomas A. Rando, Michael A. Zasloff, Kevin B. Strange, Viravuth P. Yin

**Affiliations:** 1 Kathryn W. Davis Center for Regenerative Biology and Medicine, MDI Biological Laboratory, Salisbury Cove, ME 04672 USA; 2 Department of Neurology, Stanford University Medical Center, Stanford, CA 94305-5235 USA; 3 Novo Biosciences, Bar Harbor, ME 04609 USA; 4 MedStar Georgetown Transplant Institute, Georgetown University Hospital, Washington DC, 20007 USA

## Abstract

Regenerative medicine holds substantial promise for repairing or replacing tissues and organs damaged by disease, injury, and degeneration. Much of the field has focused on development of cell-based therapeutics, gene-based therapeutics, and tissue engineering-based therapeutics. In contrast, development of small molecule regenerative medicine therapies is an emerging area. Using the adult zebrafish as a novel screening platform, we identified MSI-1436 as a first-in-class regenerative medicine drug candidate. MSI-1436 is a naturally occurring aminosterol that inhibits protein tyrosine phosphatase 1B. Treatment of adult zebrafish by intraperitoneal injection of MSI-1436 increased the rate of regeneration of the amputated caudal fin, which is comprised of bone, connective, skin, vascular and nervous tissues and also increased the rate of adult zebrafish heart regeneration. Intraperitoneal administration of MSI-1436 to adult mice for 4 weeks after induction of myocardial infarction increased survival, improved heart function, reduced infarct size, reduced ventricular wall thinning and increased cardiomyocyte proliferation. Satellite cell activation in injured mouse skeletal muscle was stimulated by MSI-1436. MSI-1436 was well tolerated by patients in Phase 1 and 1b obesity and type 2 diabetes clinical trials. Doses effective at stimulating regeneration are 5–50-times lower than the maximum well tolerated human dose. The demonstrated safety and well established pharmacological properties of MSI-1436 underscore the potential of this molecule as a novel treatment for heart attack and multiple other degenerative diseases.

## The enzyme inhibitor that regenerates multiple tissues

A naturally occurring small molecule shows promise as a drug for tissue and organ repair and regeneration. Viravuth Yin of the Kathryn W. Davis Center for Regenerative Biology and Medicine with colleagues in the US found that treating zebrafish with an intraperitoneal injection of MSI-1436, which inhibits the enzyme ‘protein tyrosine phosphatase 1B’, increased the rate of regeneration of an amputated caudal fin and of partially removed heart muscle without apparent tissue malformation. Intraperitoneal injection of MSI-1436 in adult mice also reduced the size of an induced heart infarction, improved survivability, triggered new heart muscle formation and stimulated regeneration after skeletal muscle injury. Effective doses for tissue regeneration in both animals were much lower than the maximum tolerated doses found for humans in clinical trials for potential treatment of obesity and diabetes.

## Introduction

Development of small molecules capable of activating innate tissue repair and regenerative processes is a newly emerging field in regenerative medicine.^1, 2^ Small molecules offer potential advantages over other regenerative medicine therapeutic strategies including reduced complexity and regulatory hurdles, ready reversibility of the therapy, lack of ethical concerns and likely lower treatment costs. However, small molecule discovery and development has to date been constrained by limited understanding of the molecular mechanisms underlying regenerative processes. Nevertheless, a small number of recent studies point to the potential of this approach.^3–5^

We recently undertook a phenotypic screen in zebrafish to identify small molecules capable of stimulating innate tissue repair and regeneration processes. Our screen identified the naturally occurring aminosterol MSI-1436, a potent and highly selective inhibitor of the ubiquitous protein tyrosine phosphatase 1B (PTP1B).^6, 7^ MSI-1436 was isolated originally from the liver of the dogfish shark.^8^ PTP1B dephosphorylates and inactivates receptor activated tyrosine kinases.^9, 10^

We show here that treatment of adult zebrafish with MSI-1436 stimulated the rate of regeneration of caudal fin tissue and heart muscle by 2–3-fold without apparent tissue overgrowth or malformation. Furthermore, administration of MSI-1436 to adult mice for 4 weeks beginning 24 h after inducing cardiac ischemia by permanent coronary artery ligation increased survival, improved heart function ~ 2-fold, reduced infarct size 53%, reduced ventricular wall thinning and increased cellular proliferation in the infarct border zone ~ 4-fold relative to vehicle treated control animals. Lastly, treatment of mice with MSI-1436 24 h after inducing skeletal muscle injury stimulated satellite cell activation ~ 2-fold.

MSI-1436 inhibits PTP1B via a non-competitive allosteric mechanism.^6^ In Phase 1 and 1b clinical trials as a potential treatment for obesity and diabetes, MSI-1436 was shown to be well tolerated by patients and to induce metabolic changes consistent with PTP1B inhibition.^11–13^ The doses of MSI-1436 that stimulate tissue regeneration in zebrafish and mice are 5–50-times lower than the maximum well tolerated human dose. The pharmacological properties of MSI-1436 indicate that this small molecule is a novel and promising potential regenerative medicine therapeutic for treating heart attack and other tissue damage resulting from disease and trauma.

## Results

### MSI-1436 stimulates zebrafish appendage regeneration

**Fig. 1:**
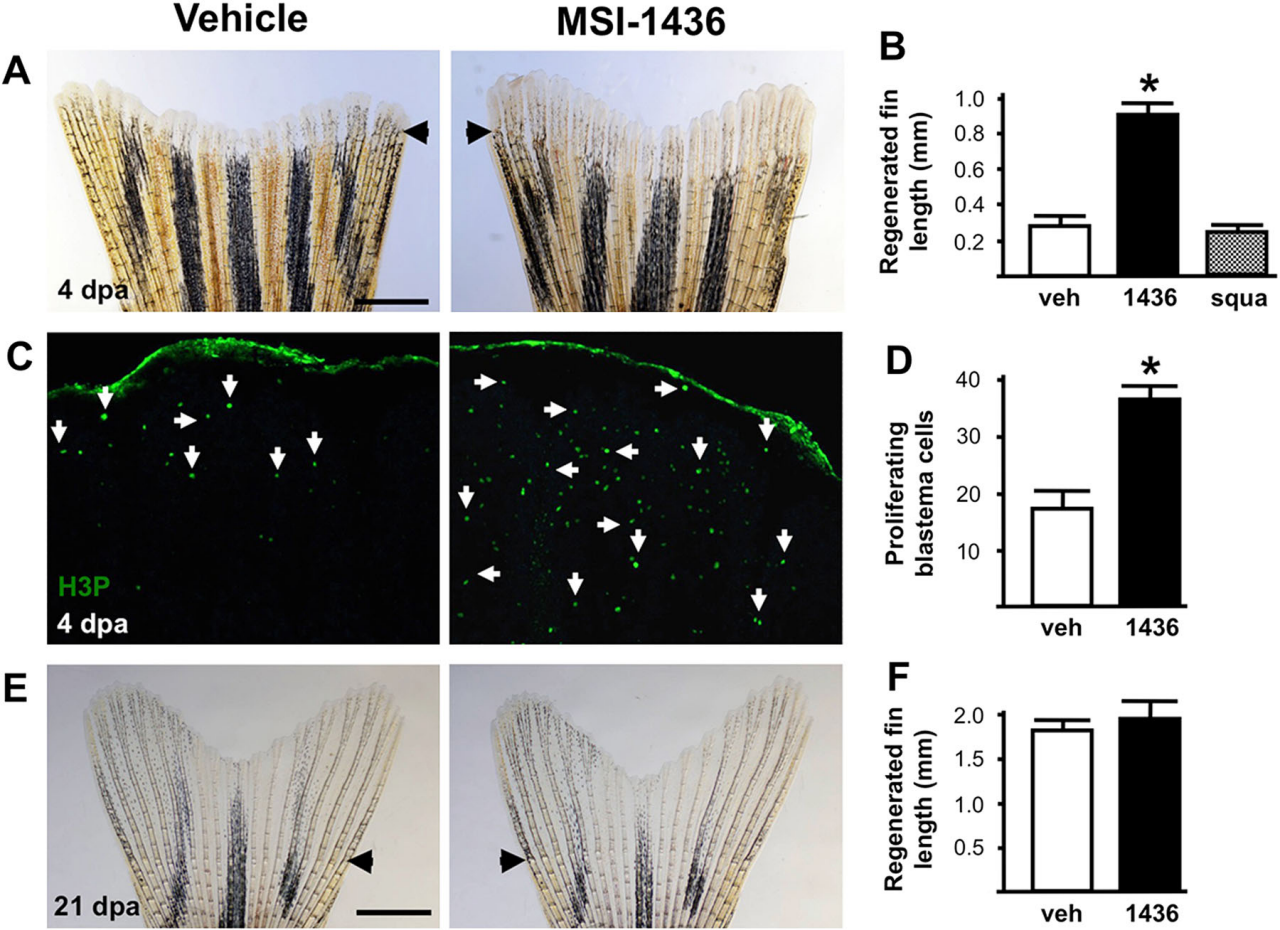
MSI-1436 stimulates adult zebrafish caudal fin regeneration. **a** Representative images of caudal fin regeneration 4 days post-amputation (dpa) in vehicle- and MSI-1436-treated fish. **b** Quantification of regenerated caudal fin length 4 dpa. Values are means ± S.E. (*n* = 12–16). *P < 0.01 compared to vehicle-treated fish. **c** Representative images of caudal fin blastemas immunostained with antibodies to phosphorylated histone 3 (H3P). Arrows show H3P-positive cells. **d** Quantification of H3P-positive cells 4 dpa. Values are means ± S.E. (*n* = 6–8). *P < 0.01 compared to vehicle-treated fish. **e** Representative images of caudal fin morphology after regeneration is complete at 21 dpa. **f** Quantification of regenerated caudal fin length 21 dpa. Values are means ± S.E. (*n* = 10). Fish were given daily given intraperitoneal (IP) injections of either vehicle, 0.125 mg/kg MSI-1436 or 0.125 mg/kg squalamine (squa) following caudal fin amputation. Arrowheads (**a**, **e**) show location of amputation plane. Scale bar (**a**, **e**) = 1 mm

The zebrafish caudal fin is comprised of bone, nerve, vasculature, connective and skin tissues, and fully regenerates within 10–14 days following amputation.^14^ The rate of caudal fin regrowth can be readily quantified. To identify small molecules capable of stimulating regeneration, adult fish were subjected to caudal fin amputation and then given daily intraperitoneal (IP) injections of either vehicle or candidate compounds. The length of regenerated caudal fin tissue was quantified at 4 days post-amputation (dpa). Treatment with 0.125 mg/kg MSI-1436 increased the rate of fin regeneration by ~ 3-fold relative to vehicle (Fig. 1a, b). No further stimulatory effect was observed at a 10-fold higher concentration (Supplementary Figure S1). MSI-1436 had no effect on caudal fin regeneration at 0.0125 mg/kg (Supplementary Figure S1).

MSI-1436 is a highly selective inhibitor of PTP1B.^6, 7^ The structurally closely related aminosterol, squalamine (Supplementary Figure S2) differs from MSI-1436 by the absence of an amine group on its polyamine tail. Importantly, squalamine had no effect on the rate of caudal fin regeneration (Fig. 1b).

Zebrafish appendage regeneration is mediated by formation of the blastema, a dedifferentiated and proliferative tissue that serves as a reservoir for proliferating cells.^15, 16^ We stained caudal fin blastemas with antibodies directed against phosphorylated histone 3 (H3P), a marker of mitosis.^17, 18^ Animals treated with MSI-1436 exhibited an ~ 2-fold increase in H3P positive cells within the blastema, suggesting that enhanced proliferation of blastemal cells could represent at least one mechanism for the increased rate of appendage regeneration (Fig. 1c, d).

Animals treated with MSI-1436 showed normal fin morphology at 21 dpa, with no signs of overgrowth or malformation (Fig. 1e, f). Moreover, zebrafish embryos and uninjured adult animals treated with daily IP injections of 1.25 mg/kg MSI-1436 for 14 and 40 days, respectively, did not exhibit obvious developmental abnormalities or altered mortality (Supplementary Fig. S3A, B). The lack of MSI-1436 toxicity in zebrafish is consistent with previous findings in mammals.^19^ Taken together, these studies suggest that MSI-1436 modulates the repair and regenerative responses of injured tissue and does not alter normal developmental processes or tissue homeostasis.

### MSI-1436 stimulates zebrafish heart regeneration

**Fig. 2:**
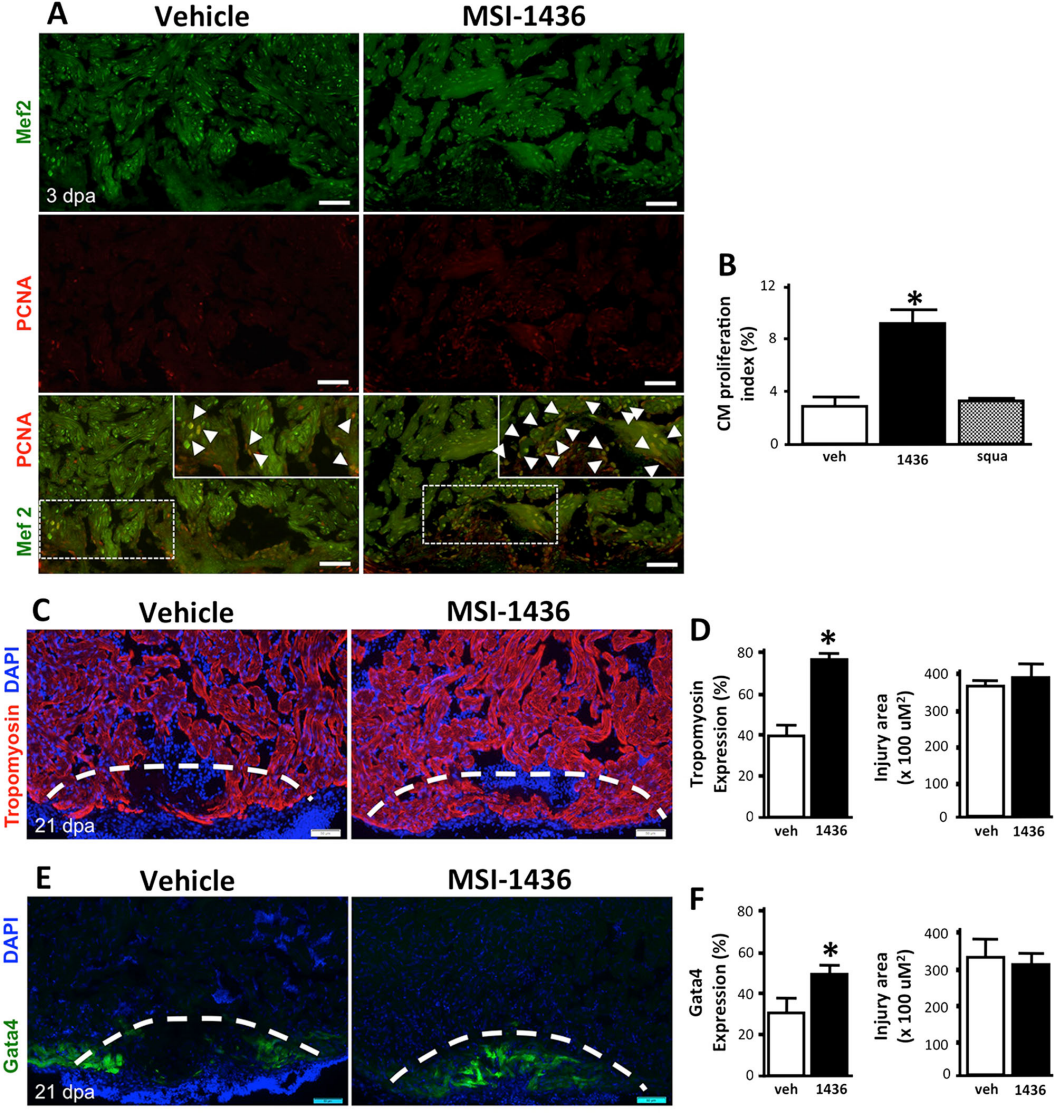
MSI-1436 stimulates adult zebrafish heart regeneration. **a** Representative images of cardiomyocyte proliferation 3 days post-amputation (dpa) of the ventricular apex in vehicle-treated fish and MSI-1436-treated fish. Arrows show proliferating cardiomyocytes expressing Mef2 and PCNA. **b** Quantification of proliferating cardiomyocytes 3 dpa expressed as percentage of cells Mef2-positive cells and PCNA-positive cells relative to cells expressing Mef2 only. Values are means ± S.E. (*n* = 10–12). *P < 0.01 compared to vehicle-treated fish. **c** Representative images of Tropomyosin expression in regenerating heart tissue 21 dpa. **d** Quantification of Tropomyosin expression and injury area 21 dpa. Values are means ± S.E. (*n* = 8–10). *P < 0.01 compared to vehicle-treated fish. **e** Representative images of *Tg(gata4:GFP)* expression in regenerating hearts at 21 dpa. **f** Quantification of *Tg(gata4:GFP)* expression and the injury area at 21 dpa. Values are means ± S.E. (*n* = 4-6). *P < 0.05 compared to vehicle-treated fish. Fish were given daily given intraperitoneal (IP) injections of either vehicle, 0.125 mg/kg MSI-1436 or 0.125 mg/kg squalamine (squa) following ventricular resection. Dashed lines show approximate resection plane

Given the stimulatory effects of MSI-1436 on the regenerative response of the caudal fin, we also tested its effect on heart regeneration. Adult zebrafish were subjected to a partial ventricular resection, removing ~ 20% of the ventricular mass, and subsequently given daily IP injections of either vehicle or MSI-1436 at a concentration of 0.125 mg/kg for 3 days post-surgery. We first assessed CM proliferation indices as a metric for regenerative capacity. Hearts were isolated at 3 dpa, cryosectioned and stained to identify cells co-expressing the CM marker Mef2 and the proliferation marker proliferating cell nuclear antigen (PCNA) (Fig. 2a). To establish a CM proliferation index, we quantified Mef2-positive cells and PCNA-positive cells as a percentage of the total number of cells expressing only Mef2 within a defined injury area.^20^ Relative to vehicle control, MSI-1436 treatment increased CM proliferation ~ 2.6-fold at 3 dpa (Fig. 2a, b). Similar to appendage regeneration, squalamine had no effect on CM proliferation (Fig. 2b). Vivo-morpholino (MO) knockdown of PTP1B in adult zebrafish had a similar effect to that of MSI-1436 on CM proliferation (Supplementary Fig. S4A, B). Combinatorial treatments with PTP1B MO and MSI-1436 did not significantly elevate CM proliferation in comparison to PTP1B MO treatment alone (Supplementary Fig. S4B), suggesting MSI-1436 stimulation is mediated by repression of PTP1B activity.

Regeneration of the zebrafish heart is complete within 2 months following ventricular resection.^21^ To determine whether MSI-1436 increases the rate of heart regeneration, we quantified the expression of Tropomyosin, a muscle specific marker expressed in differentiated cardiac sarcomeres, by immunohistochemistry.^22^ MSI-1436 treatment increased Tropomyosin expression nearly 2-fold within the injury zone at 21 dpa (Fig. 2c, d). To confirm these findings, we employed the *Tg(gata4:GFP)* reporter strain.^23^ At 21 dpa, *Tg(gata4:GFP)* is expressed in the outer muscle layer of the heart and in newly regenerated muscle within the injury environment. Treatment of injured hearts with MSI-1436 stimulated an ~ 1.5-fold increase in *Tg(gata4:GFP)* expression within the injury zone in comparison to vehicle treatment (Fig. 2e, f). Thus, by two independent analyses, we demonstrate that MSI-1436 administration results in an increase in regenerated muscle. Taken together, data in Fig. 2 demonstrate that MSI-1436 is potent stimulator of zebrafish CM proliferation and new heart muscle regeneration following acute heart injury.

### MSI-1436 stimulates recovery of heart function after ischemic injury in the adult mouse

The neonatal mouse heart regenerates in a manner similar to that of the adult zebrafish. However, the capacity for heart regeneration in mice is lost rapidly approximately one week after birth.^24^ Given the striking effect of MSI-1436 on regeneration of multiple tissue types in adult zebrafish, we examined the effect of this compound on heart injury in the adult mouse, which has limited heart regenerative capacity.^25, 26^ Ischemic heart injury was induced in 6–8 week old mice by permanent ligation of the left anterior descending (LAD) coronary artery.^27^ Twenty-four hours after ligation, we performed echocardiography to confirm the presence of heart injury. These mice were then administered MSI-1436, at either 0.125 or 1.25 mg/kg, or vehicle only, via IP injections. Based on the known pharmacokinetics of MSI-1436 in mice,^19^ injections were repeated every 3 days to maintain plasma concentration levels.

**Fig. 3:**
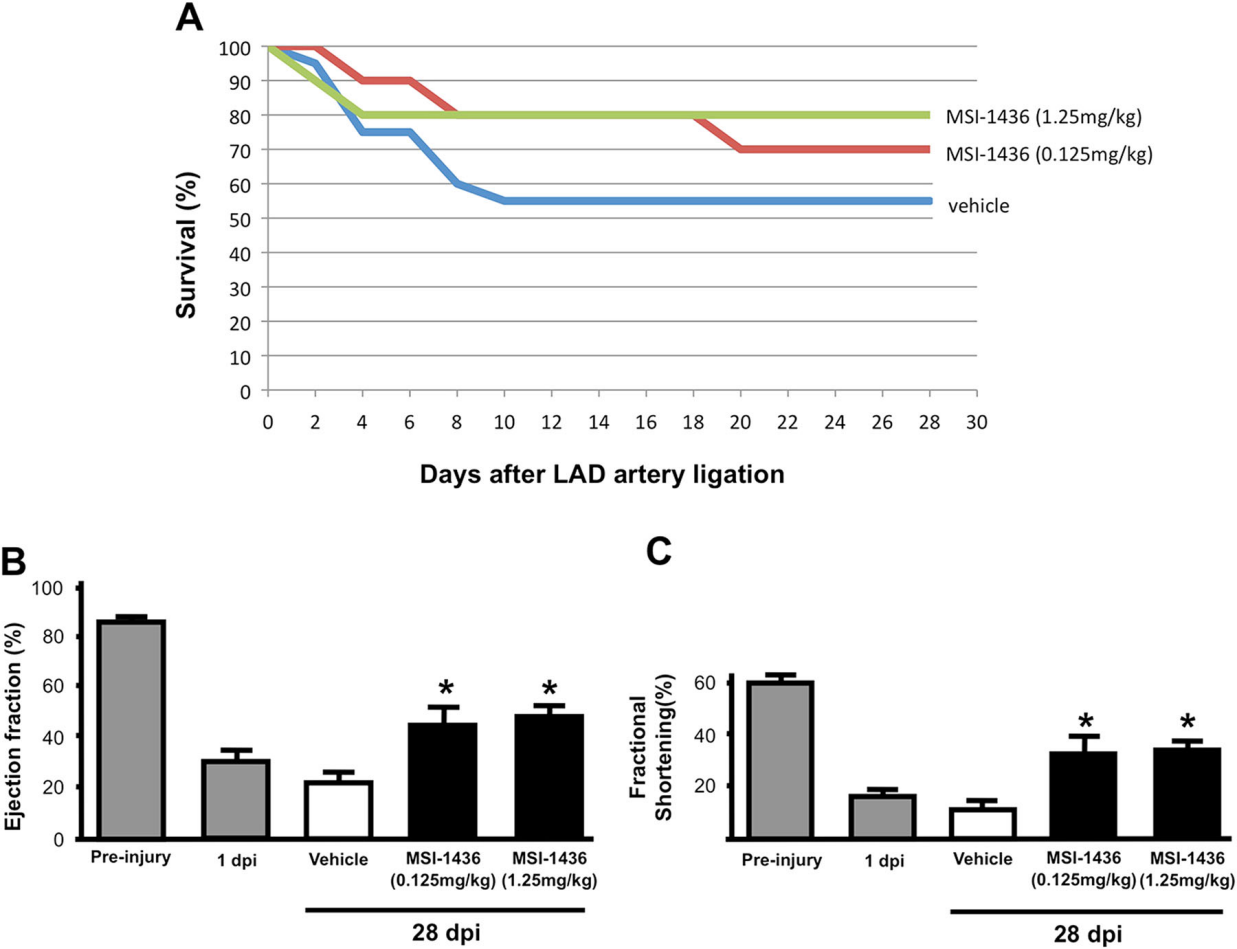
MSI-1436 improves survival and heart function in adult mice following ischemic injury. **a** Survival in mice treated with vehicle, 0.125 mg/kg MSI-1436 or 1.25 mg/kg MSI-1436. *n* = 10–20 mice in each treatment group. Ejection fraction **b** and fractional shortening **c** as measured by echocardiography. Values are means ± S.E. (*n* = 10-18). *P < 0.05 compared to vehicle-treated mice. Mice were administered intraperitoneal (IP) injections of either vehicle, 0.125 mg/kg MSI-1436 or 1.25 mg/kg MSI-1436 beginning 24 h after LAD artery ligation

MSI-1436 administration increased survival at 28 days from 55% (*n* = 20) in vehicle-treated control animals to 70 and 80% (*n* = 10; 20) in mice administered 0.125 or 1.25 mg/kg MSI-1436, respectively (Fig. 3a).

To determine the effects of MSI-1436 treatment on heart function, we performed echocardiography at 4 weeks after LAD artery ligation to quantify changes in fractional shortening and ejection fraction. As expected, at 1-day post-injury (dpi) and prior to drug treatment, all animals showed significantly reduced heart function when compared to baseline values of uninjured animals (Fig. 3b, c). However, at 28 dpi, both fractional shortening and ejection fraction exhibited a ~ 2–3-fold improvement in MSI-1436 treated animals compared to vehicle controls (Fig. 3b, c; Supplementary Table S1). No difference in the degree of improvement was observed in mice administered 0.125 vs. 1.25 mg/kg MSI-1436.

### MSI-1436 treatment reduces infarct size and stimulates cell cycle entry in adult mouse heart

**Fig. 4:**
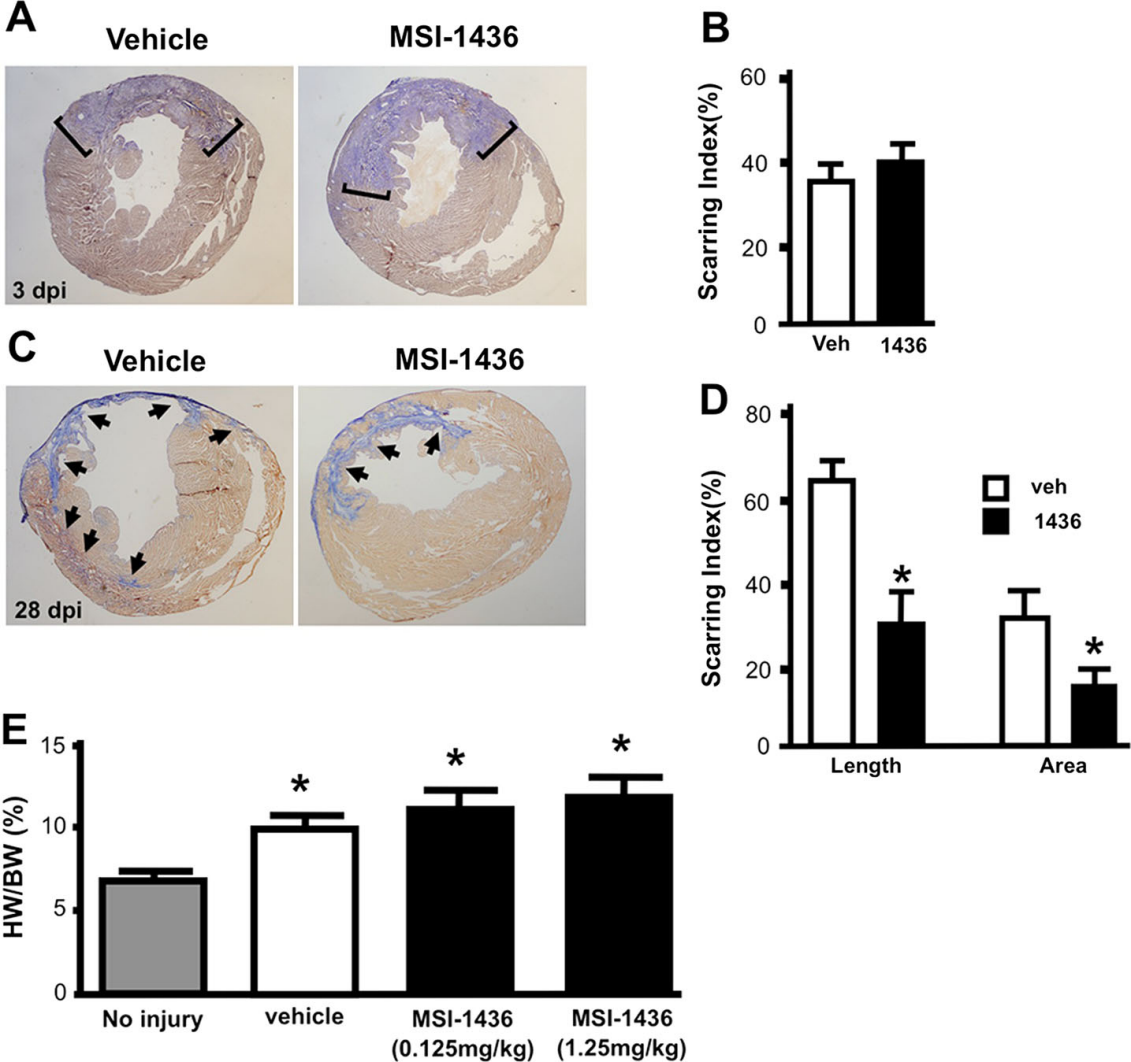
MSI-1436 reduces infarct size in adult mouse heart following ischemic injury. **a**, **b** Representative images of infarcts and quantification of infarct size in hearts isolated 3 days post-injury from mice treated with vehicle or 0.125 mg/kg MSI-1436. Brackets demarcate collagen deposition. Values in (**b**) are means ± S.E. (*n* = 8-10). **c**, **d** Representative images of infarcts and quantification of infarct size in hearts isolated 28 days post-injury from mice treated with vehicle or 0.125 mg/kg MSI-1436. Arrows indicate scar tissue in the left ventricle. Values in (**d**) are means ± S.E. (*n* = 8). *P < 0.05 compared to vehicle-treated mice. **e** Heart weight/body weight ratio in uninjured mice and mice 28 dpi treated with vehicle or MSI-1436. *P < 0.05 compared to vehicle-treated mice (*n* = 7–16)

To determine how MSI-1436 improved heart function, we performed a series of histological studies. Infarct scar size in hearts isolated 3 dpi was similar in vehicle- and MSI-1436-treated mice (Fig. 4a, b) indicating that administration of MSI-1436 did not reduce the initial amount of ischemic damage and scar formation. However, infarct scar size was reduced 53% in mice administered 0.125 mg/kg MSI-1436 for 4 weeks compared to untreated mice (Fig. 4c, d; Supplementary Fig. S5). Quantification of scar tissue by area measurements and infarct length yielded similar levels of decrease in scar tissue (Fig. 4d).

Thinning of the ventricular wall was also greatly reduced in animals administered MSI-1436 (Fig. 4c). Hearts isolated 28 dpi from mice administered vehicle or MSI-1436 showed similar increases in heart weight to body weight ratio (HW/BW) (Fig. 4e; Supplementary Table S2) indicating the presence of hypertrophy and/or new muscle formation. Previous MSI-1436 studies on mice demonstrated a dose-dependent decrease in body weight.^19^ However, drug treatment at 0.125 and 1.25 mg/kg did not induce significant changes to body weight at 4 weeks, suggesting the increase in HW/BW is not due to weight loss (Supplementary Table S2).

**Fig. 5:**
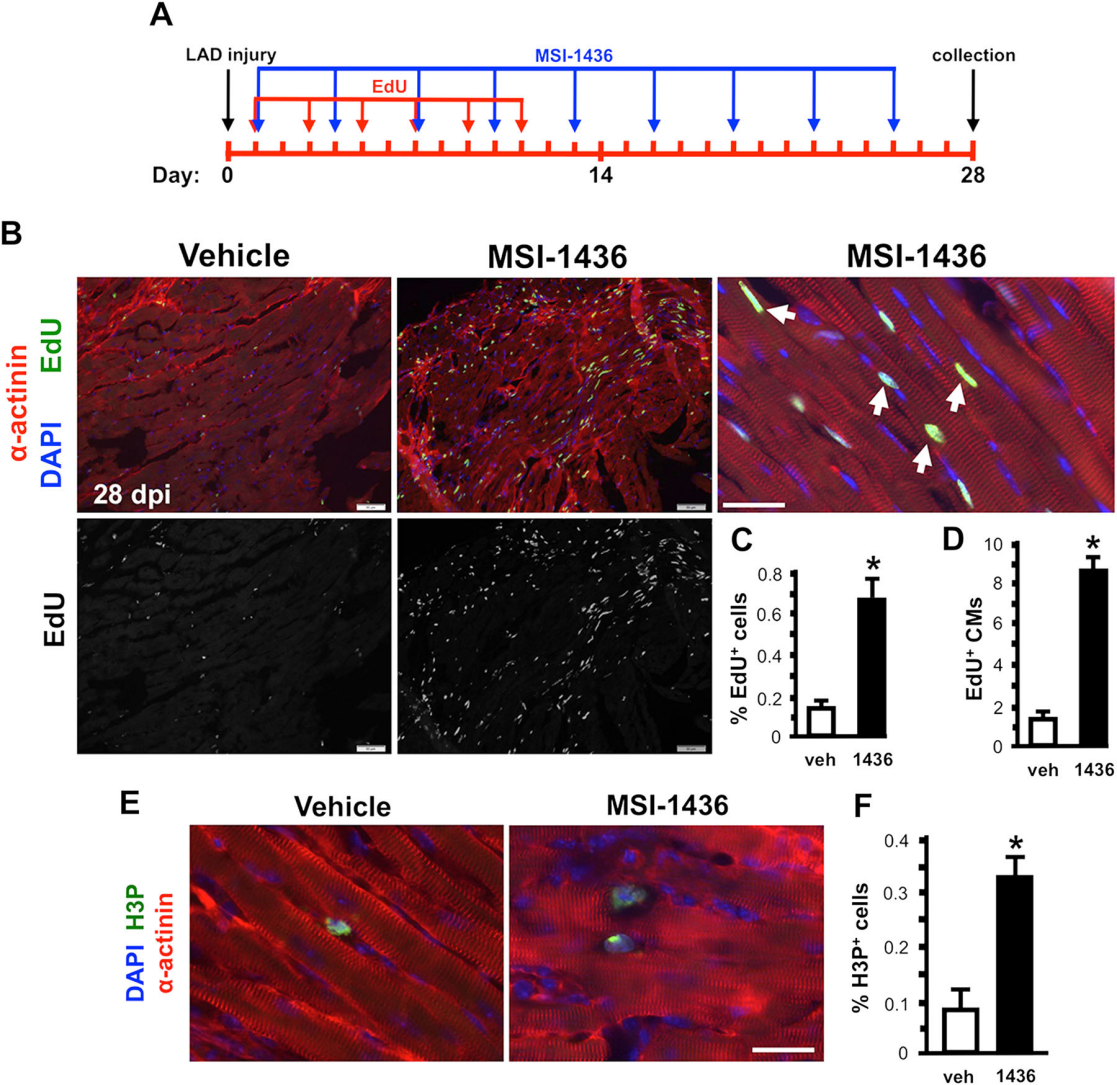
MSI-1436 stimulates cardiomyocyte proliferation in adult mouse heart following ischemic injury. **a** Protocol for EdU labeling of mouse hearts. **b** Representative images showing EdU labeling of infract border zone in hearts isolated 28 dpi from vehicle-treated mice and mice administered 0.125 mg/kg MSI-1436. Arrowheads indicate EdU+ cardiomyocytes. Scale bar in left and middle panels corresponds to 50 microns. Scale bar in right panel is 25 microns. **c**, **d** Quantification of EdU labeled cells and EdU+ cardiomyocytes (CMs). Values are means ± S.E. (*n* = 8). *P < 0.05 compared to vehicle-treated mice. **e** Representative image of cardiomyocytes labeled with antibodies to phosphorylated histone 3 (H3P). **f** Values are means ± S.E. (*n* = 5-7). *P < 0.05 compared to vehicle-treated mice. Scale bar in **(e)** is 25 microns

To determine whether reduced infarct scar size and increased heart weight were associated with increased cellular proliferation, we quantified EdU labeling in hearts isolated 28 days after LAD artery ligation. EdU labeling in the infarct border zone was increased 4.5-fold by MSI-1436 treatment (Fig. 5b, c). Similar results were observed using antibodies directed against H3P (Fig. 5e, f). A sub-population of the EdU labeled cells expressed sarcomeric α-actinin, a marker of mature cardiomyocytes (Fig. 5B). MSI-1436 treatment increased the number of EdU and sarcomeric α-actinin double positive cardiomyocytes 5.7-fold from an average of 1.5 cells in vehicle to 8.7 cells in drug treated hearts (Fig. 5D). EdU labeling in regions distant from the infarct border zone was nominal and no differences were observed between vehicle- and MSI-1436-treated mice (Supplementary Fig. S6).

### MSI-1436 administration activates mouse skeletal muscle satellite cell proliferation

Caudal fin and heart regeneration in adult zebrafish^14, 28^ occurs through a primary mechanism of dedifferentiation of spared cells within the injury zone. To determine whether MSI-1436 also acts on stem cells per se, we quantified the proliferative activity of Pax7-expressing skeletal muscle satellite cells, which are stem cells essential for muscle regeneration.^29, 30^

**Fig. 6:**
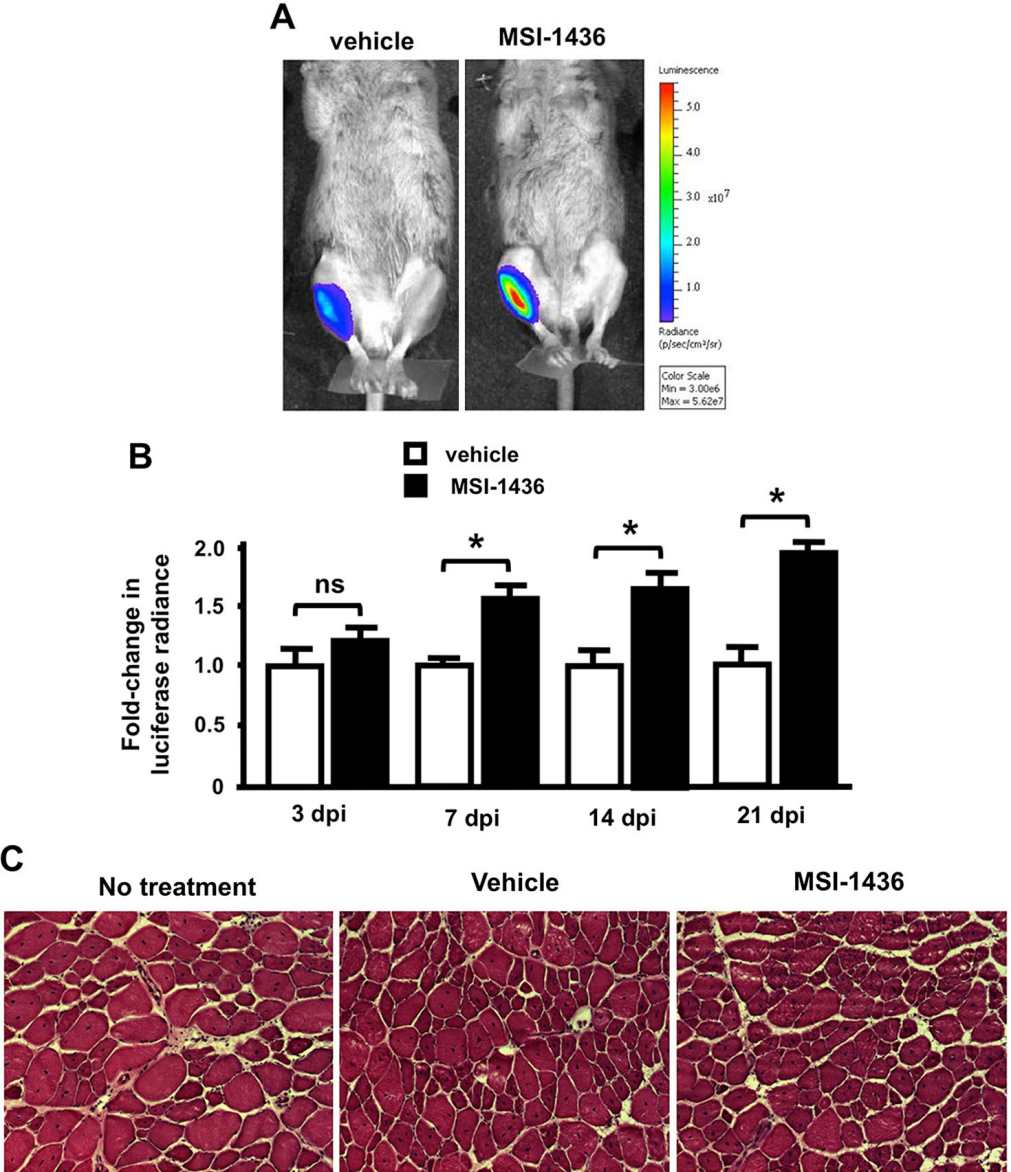
MSI-1436 stimulates satellite cell proliferation in injured mouse skeletal muscle. **a** Representative images showing luciferase signals from injured limbs of mice treated with vehicle or 0.125 mg/kg MSI-1436. The color scale represents photon emission from the tissue surface and is expressed as radiance (p/sec/cm^2^/sr). **b** Relative luciferase radiance 3-21 days post-injury (dpi) in vehicle-treated mice and mice administered 0.125 mg/kg MSI-1436. Values are means ± S.E. (*n* = 4). *P < 0.05 compared to vehicle-treated mice. **c** Representative images showing muscle morphology at 21 dpi in mice with no treatment and mice treated with vehicle or 0.125 mg/kg MSI-1436. Mice were administered intraperitoneal (IP) injections of either vehicle or 0.125 mg/kg MSI-1436 beginning 24 h after injury of the anterior tibialis muscle by BaCl_2_ injection

Pax7 is a well-defined marker of the proliferative activity of satellite cells and can be monitored noninvasively using mice engineered to express a Pax7-driven luciferase reporter (*Pax7CreER/LuSEAP*).^31, 32^ To determine whether MSI-1436 alters satellite cell proliferative activity, we injured tibialis anterior muscles of *Pax7CreER/LuSEAP* mice by BaCl_2_ injection. Twenty-four hours after injury, mice were administered by IP injection vehicle or 0.125 mg/kg MSI-1436. Injections were repeated every three days. MSI-1436 increased reporter activity 1.5-2-fold 7–21 days after injury was induced (Fig. 6a, b). At 21 days after injury when muscle regeneration is complete, muscle morphology was similar in vehicle-treated mice and MSI-1436-treated mice (Fig. 6c), indicating that MSI-1436 increases satellite cell proliferation without inducing aberrant tissue regeneration.

## Discussion

We have shown that MSI-1436 increases CM proliferation and the rate of heart regeneration in adult zebrafish (Fig. 2). Administration of MSI-1436 to adult mice, which have little capacity for heart regeneration,^25^ reduces mortality, improves heart function 2–3-fold, reduces infarct size 53% and reduces ventricular wall thinning (Figs. 3, 4). MSI-1436 induces a 5.7-fold increase in cardiomyocyte proliferation in the infarct border zone (Fig. 5). These results indicate that reduced infarct size and ventricular wall thinning and improved heart function observed in adult mice is due at least in part to stimulation of heart regeneration by MSI-1436.

MSI-1436 also strikingly increases the rate of regeneration of zebrafish caudal fin, an appendage comprised of multiple interacting tissue types (Fig. 1), and it stimulates satellite cell proliferation in injured mouse skeletal muscle (Fig. 6). These results suggest that MSI-1436 could be used to speed wound healing and induce the repair and regeneration of multiple organ and tissue types. Consistent with this idea, PTP1B knockout in mice has been shown to stimulate the repair of diverse tissues,^33–35^ improve heart function after MI^36, 37^ and improve cardiovascular function during aging.^36^ Importantly, MSI-1436 crosses the blood-brain barrier^7, 38^ and inhibits PTP1B in the CNS.^39^ It is thus conceivable that systemic administration of MSI-1436 could possibly be used to treat brain injuries.

The exact cell type(s) and cellular processes on which MSI-1436 is acting to enhance and induce regeneration are unclear at present. PTP1B is ubiquitously expressed and functions to dephosphorylate and inactivate receptor tyrosine kinases (RTK) that have been activated by ligand binding-induced autophosphorylation.^9, 10^ RTK signaling plays critical roles in cell metabolism, growth, differentiation and survival and regenerative and wound repair processes.^9, 10, 40, 41^ Inhibition of PTP1B is thus expected to enhance the activity of multiple pro-regenerative signaling processes.

In zebrafish, MSI-1436 clearly increases the number of proliferating CMs (Fig. 2b) and blastema cells (Fig. 1d). MSI-1436 strikingly increases the number of proliferating cells in the ischemic adult mouse heart (Fig. 5c, f). Enhanced proliferation could be due to a stimulatory effect of MSI-1436 on dedifferentiation of precursor cells and/or stimulation of cell division. Increased proliferation of satellite cells by MSI-1436 in injured mouse skeletal muscle (Fig. 6b) indicates that PTP1B inhibition increases stem cell division per se. Our studies, however, do not distinguish between whether Pax7 expression is increased or whether there are more Pax7 expressing cells. Future mechanistic studies are required to ascertain the mechanism of action on MSI-1436 on skeletal muscle. PTP1B inhibition may also increase proliferation and regeneration by reducing cell death.^42–44^

A growing body of evidence indicates that the immune system plays a critical role in modulating regenerative processes.^45^ Immune cells clear cellular debris and secrete factors that modulate progenitor cell activation and stem cell function. They also secrete inflammatory factors that can cause further tissue damage. In addition to acting directly on regenerating cell and tissue types, MSI-1436 could also enhance pro-regenerative immune system functions. For example, PTP1B inhibition reduces cardiovascular inflammation during sepsis,^46^ protects against inflammation-induced gliosis in the retina^47^ and reduces brain inflammation induced by LPS injection.^48^ Defining the specific pro-regenerative mechanisms that are modulated by MSI-1436 will clearly require extensive additional research.

PTP1B has been a major pharmaceutical target for possible treatment of type 2 diabetes, obesity and cancer. Numerous PTP1B inhibitors have been developed. Only one of these, ertiprotafib, was deemed to have properties appropriate for testing in human, but was abandoned in Phase II clinical trials because of off-target effects and efficacy concerns.^49^ MSI-1436 has been tested in Phase 1 and 1b clinical trials as a possible treatment for obesity and type-2 diabetes. Data consistent with inhibition of PTP1B were reported and the molecule was shown to be well tolerated by patients.^11–13^ Importantly, the doses we have shown to be effective in stimulating tissue regeneration are 5–50-times lower than the maximum well-tolerated human dose.

It is particularly noteworthy that MSI-1436 stimulates tissue regeneration in both zebrafish and adult mice, two widely divergent species that are separated by approximately 450 million years of evolution. Equally noteworthy are the observations that MSI-1436 stimulates regeneration of diverse tissues types without inducing tissue malformation (Figs. 1, 2, 4 and 6) or disrupting sensitive developmental processes (Supplementary Fig. S3). The demonstrated safety and efficacy of MSI-1436 in humans, its effect on regeneration in animals as divergent as mice and zebrafish and on different tissue types, combined with extensive knowledge of its target underscore the potential of this molecule as a treatment for multiple human diseases in which stimulation of tissue repair and regeneration is an optimal outcome.

## Materials and methods

### Study design

All drug treatment studies in both zebrafish and mice were carried out in a blinded and randomized fashion. Analysis of histological images was also done blinded and randomized. All animal studies were pre-approved by the Institutional Animal Care and Use Committee (IACUC) at MDI Biological Laboratory, Cardio-Lab or Stanford University prior to being performed. Drugs were administered via IP microinjections either immediately following adult zebrafish amputation or at 24 h post injury in adult mice.

### Adult zebrafish amputations

Zebrafish were maintained at 27 °C on a 14 h light/10 h dark cycle in accordance to IACUC protocols at the MDI Biological Laboratory. Adult 6–9 month old zebrafish of the Ekkwill (EK) strain were anesthetized in 2- phenoxyethanol (1:1000) to effect and subjected to injury. Caudal fins were amputated at 50% length, as determined from the basal body to the distal tip of the caudal fin. Apical ventricular resection surgeries were performed as previously described.^21^ In brief, ~ 20% of the ventricle was removed with iridectomy scissors and animals were allowed to recover in system fish water following surgery. Hearts were then extracted at the desired stage for examination.

### Adult mouse myocardial infarction studies

These studies were performed by Cardio-Lab (http://cardio-lab.com/), a contract research organization. Adult C57BL/6J mice weighing between 22–25 g were anesthetized using 2% isoflurane and 100% oxygen at a flow rate of 2.5 L/min. To induce myocardial ischemia, the LAD coronary artery was permanently ligated using well established protocols.^27^ Briefly, the left coronary artery was ligated with a 7–0 suture at about 2 mm below the left auricle to induce a permanent occlusion. Ischemia was confirmed by ST-segment elevation using a Power Lab/4SP with ML135 Dual Bio Amp electrocardiogram system and decreased coloration of heart muscle. After closure of the chest and resumption of spontaneous respiration, animals were returned to their cages and provided with standard chow and water. Twenty-four hours after surgery, transthoracic echocardiography was performed using an Acuson P300 with 18 MHz transducer. Animals with compromised heart function were then administered by IP injection vehicle or MSI-1436. Injections were repeated every 3 days to maintain plasma drug concentrations.

### Adult mouse hindlimb injury

*Pax7Cre* ^ER^ *LuSEAP* mice were anesthetized using 2% isoflurane and 100% oxygen at a flow rate of 2.5 L/min. Twenty-five microliters of a 1.2% BaCl_2_ solution was injected directly in to the tibialis anterior muscle using a 10 cc syringe. Twenty-four hours after the injury, mice were administered by IP injection vehicle or 0.125 mg/kg MSI-1436. Injections were repeated every 3 days to maintain plasma drug concentrations.

Beginning at 3 days post-injury, bioluminescent imaging was performed using a Xenogen IVIS-Spectrum system (Caliper Life Sciences). Mice were again anesthetized. Three hundred microliters of a 50 mg/mL sterile D-Luciferin firefly substrate (Biosynth International, Inc.) dissolved in PBS were administered by IP injection and 23 min after substrate injection the mice were imaged for 30 s at the maximal light collection (f-stop 1) at the highest resolution (small binning). Each image was saved for subsequent analysis.

### Histological methods

5-ethynyl-2′deoxyuridine (EdU) (15 mg/kg) (www.invitrogen.com) was IP microinjected every other day beginning at 1 day post-injury for a total of 6 injections (Fig. 5).

Zebrafish and mouse hearts were extracted and fixed in 4% paraformaldehyde (PFA), embedded in tissue freezing medium (Fisher) and sectioned at 10 and 6 μm with Leica CM1860 cryostat, respectively. Immunofluorescence and acid fuchsin orange G (AFOG) stains were performed as previously described.^20, 21^ We used the following primary antibodies: anti-Mef2 (rabbit; Santa Cruz Biotechnology #SC-313; 1:75), anti-PCNA (mouse; Sigma #P8825; 1:400), anti-H3P (Millipore #06-570) and anti-sarcomeric α-actinin (Abcam #AB9465). EdU was detected with Click-iT® EdU Alexa Fluor® 488 Imaging Kit (C10337) in accordance to manufacturer’s instructions.

For mouse skeletal muscle, hindlimb muscles were submerged in 0.5% PFA for 5 h and then dehydrated in 20% sucrose overnight at 4 °C. The muscles were then submerged in Optimal Cutting Temperature (OCT) Compound (Sakura Finetek USA, Inc.) and frozen for approximately 30 s in liquid nitrogen chilled in isopentane. Muscle cryosections (6 μm thick) were placed on coated glass slides and processed for hematoxylin and eosin staining as previously described.

Zebrafish cardiomyocyte (CM) proliferation indices were defined as the total number of Mef2+PCNA+ cells represented as a percentage of the total Mef2+ population. Briefly, Mef2+/PCNA+ and Mef2+ cells were manually counted within a defined region of 230 pixels. Three sections containing the largest injury area were quantified for at least 5 hearts in each treatment group.

To identify the injury site at 21 dpa, we examined natural autofluorescence in myocardial tissue to demarcate the amputation plane. While uninjured muscle has strong autofluorescence, autofluorescence is much weaker in regenerating muscle. The intersection between high and low autofluorescence was used to define the approximate amputation plane.^22^

Quantification of adult mouse cell proliferation was performed using EdU labeling or by immunolabeling with anti-H3P antibodies. Labeling was assessed in 5–8 consecutive serial sections beginning with the largest infarct section. EdU- and H3P-positive cells were manually counted within a 20X field of view.^24, 50^

To quantify scar tissue, mouse hearts were cryosectioned at a thickness of 6 μm, with an interval of 300 μm between each section, and stained with AFOG. Scarring indices were determined by measurement of both infarct area and infarct length. To determine infarct area, we used Photoshop CS6 to outline the infarct zone area and the entire left ventricle for each section of each heart. The scarring index is represented as a ratio of the infarct area sum over the sum of entire area of the left ventricle. To quantify infarct length, the length for epicardium infarct, the length of the endocardium infarct, the epicardium circumference and the endocardium circumference was traced with Photoshop CS6. The infarct length was calculated according to established formulas.^51^ Scarring was quantified in at least 8 sections per heart.

### Intraperitoneal microinjections

Antisense vivo-morpholino directed against *PTP1B* (5′ CGATTTCCCGAAACTCGGCTTCCAT 3′) was injected daily at 1.0 mg/kg body weight for the duration of the experiment (www.Gene-Tools.com). Ventricular resection was performed on day 2 of injections.

### Statistical analysis

All statistics were performed using Student’s t-test with Welch’s correction. A *P*-value < 0.05 was deemed statistically significant.

## Electronic supplementary material

Supplementary Information

Supplementary Figure S1

Supplementary Figure S2

Supplementary Figure S3

Supplementary Figure S4

Supplementary Figure S5

Supplementary Figure S6

Supplementary Table S1

Supplementary Table S2
