# Supplementary material for: The protein tyrosine phosphatase 1B inhibitor MSI-1436 stimulates regeneration of heart and multiple other tissues
Source: NPJ Regen Med. 2017 Mar 3;2:4. doi: 10.1038/s41536-017-0008-1 (PMC5677970; doi:10.1038/s41536-017-0008-1)
Supplement: Supplementary file 1 — Supplementary Information [file 41536_2017_8_MOESM1_ESM.docx]

**SUPPLEMENTARY MATERIALS**

**Supplementary Figure S1. Dose dependent response of caudal fin regeneration to intraperitoneal injections of MSI-1436 for 4 days post-amputation.** Values are means ± S.E. (n=6-10). *P<0.01 compared to vehicle-treated fish.

**Supplementary Figure S2. Chemical structures of MSI-1436 and squalamine.**

**Supplementary Figure S3. Effect of MSI-1436 on survival of developing zebrafish embryos and adult zebrafish.** One-cell zebrafish embryos (**A**) or 3-month old adult zebrafish (**B**) were administered daily intraperitoneal injections of vehicle or 1.25 mg/kg MSI-1436. Embryos developed normally and showed no obvious gross morphological or behavioral abnormalities. n = 323 embryos and 28 adult zebrafish.

**Supplementary Figure S4. Antisense oligonucleotide mediated depletion of PTP1B enhances cardiomyocyte proliferation.** (**A**) Representative images of cardiomyocyte proliferation 3 days post-amputation (dpa) of the ventricular apex in adult zebrafish treated with a scrambled control morpholino (MO) or a MO directed against PTP1B. Arrows show proliferating cardiomyocytes expressing Mef2 and PCNA. (**B**) Quantification of proliferating cardiomyocytes 3 dpa expressed as percentage of Mef2+PCNA+ cells relative to cells expressing Mef2 only. Values are means ± S.E. (n=9). *P<0.01 compared to control fish. Adult zebrafish were treated with daily intraperitoneal microinjections of scrambled control or PTP1B MO (1mg/kg) or PTP1B MO with MSI-1436.

**Supplementary Figure S5. Effects of MSI-1436 on scar tissue formation.** Representative heart sections from vehicle and MSI-1436 (0.125mg/kg) treated hearts were stained with acid fuchsin orange G staining to detect collagen (blue) and muscle (brown).

**Supplementary Figure S6. Effect of MSI-1436 on cell proliferation in remote region from the infarct.** Representative images showing EdU labeling of remote zone in hearts isolated 28 dpi from vehicle-treated mice and mice administered 0.125 mg/kg MSI-1436. Scale bar corresponds to 50 microns.

**Supplementary Table S1. Echocardiography measurements in vehicle and MSI-1436 treated animals.** Echocardiography measurements were performed prior to, 1 day post- and 28 days post-MI. Fractional shortening (FS) and ejection fraction (EF) values are shown for each animal.

**Supplementary Table S2. Body weight (g) of vehicle and MSI-1436 treated mice at 28 days post-MI.**
