# Supplementary figures and images for: The protein tyrosine phosphatase 1B inhibitor MSI-1436 stimulates regeneration of heart and multiple other tissues
Source: NPJ Regen Med. 2017 Mar 3;2:4. doi: 10.1038/s41536-017-0008-1 (PMC5677970; doi:10.1038/s41536-017-0008-1)

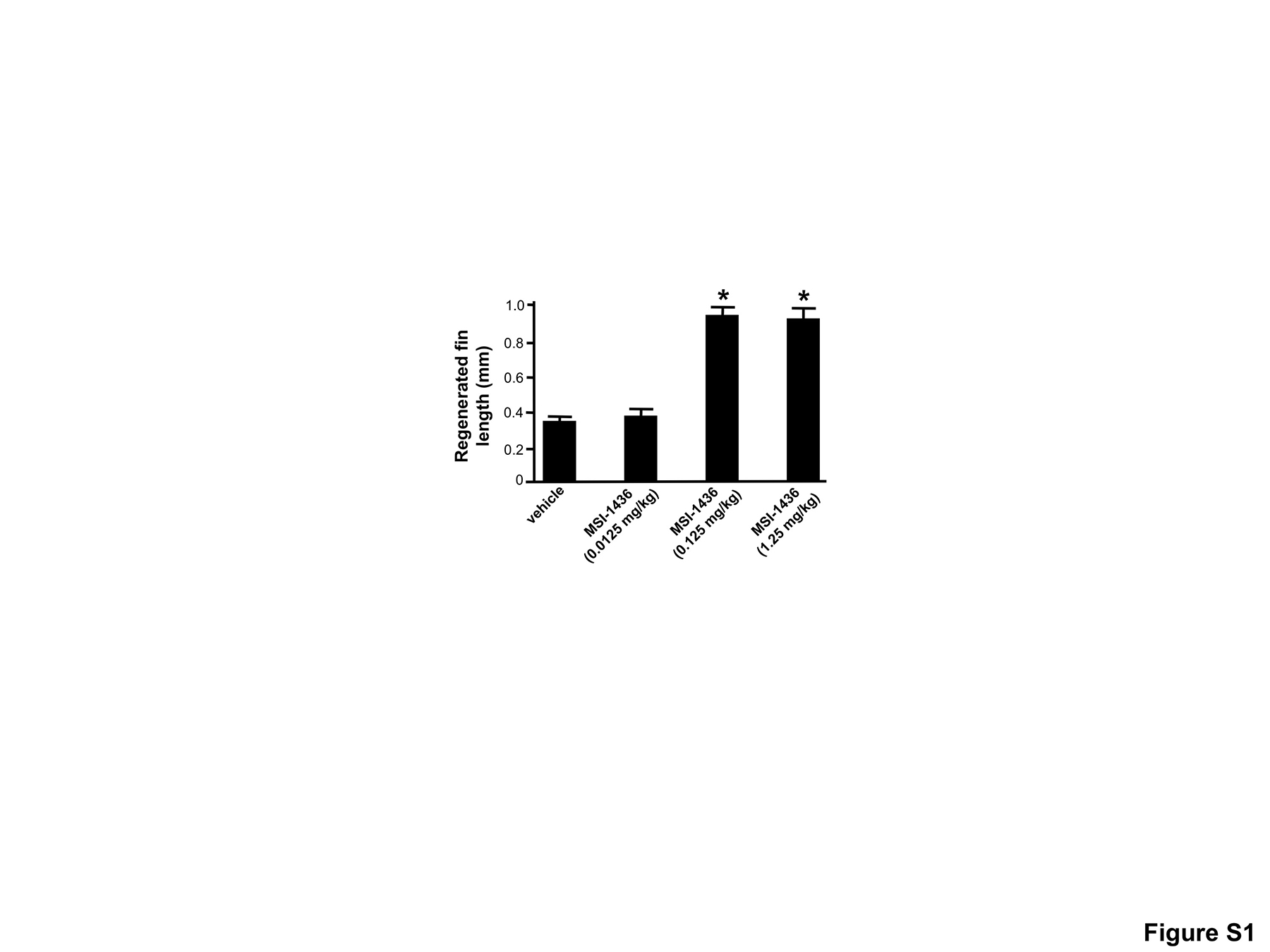

Supplement: Supplementary file 2 — Supplementary Figure S1 [file 41536_2017_8_MOESM2_ESM.jpg]

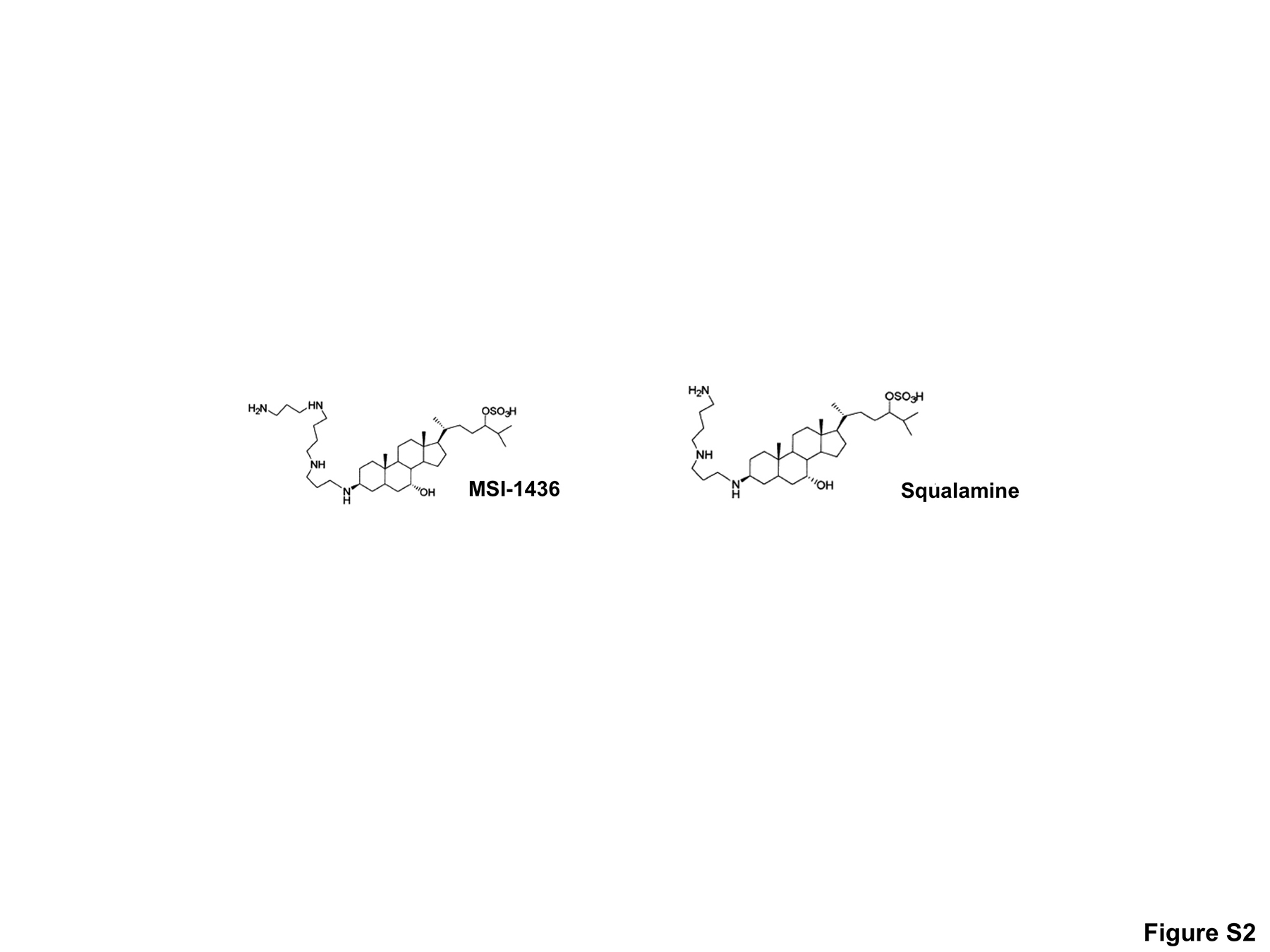

Supplement: Supplementary file 3 — Supplementary Figure S2 [file 41536_2017_8_MOESM3_ESM.jpg]

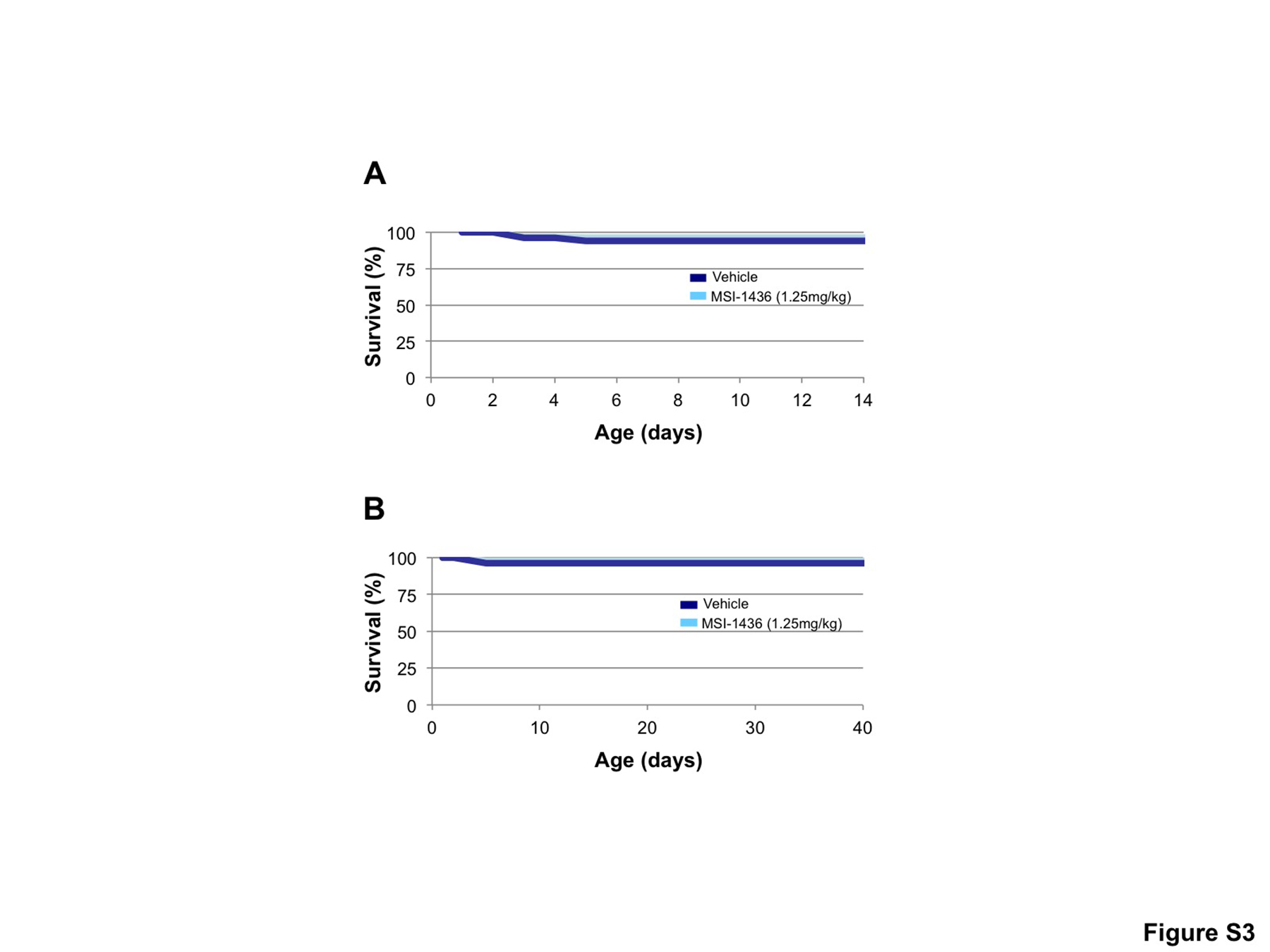

Supplement: Supplementary file 4 — Supplementary Figure S3 [file 41536_2017_8_MOESM4_ESM.jpg]

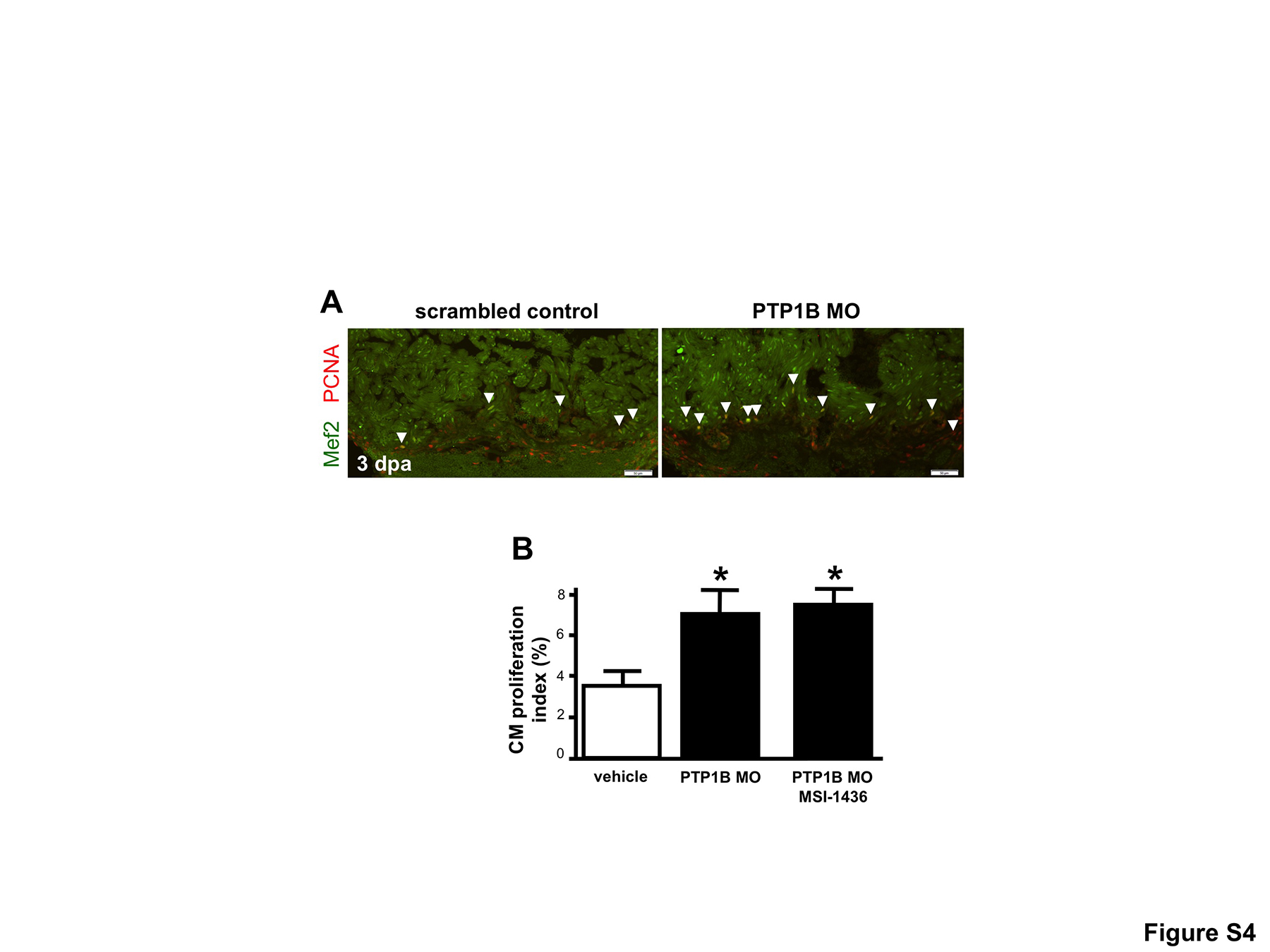

Supplement: Supplementary file 5 — Supplementary Figure S4 [file 41536_2017_8_MOESM5_ESM.jpg]

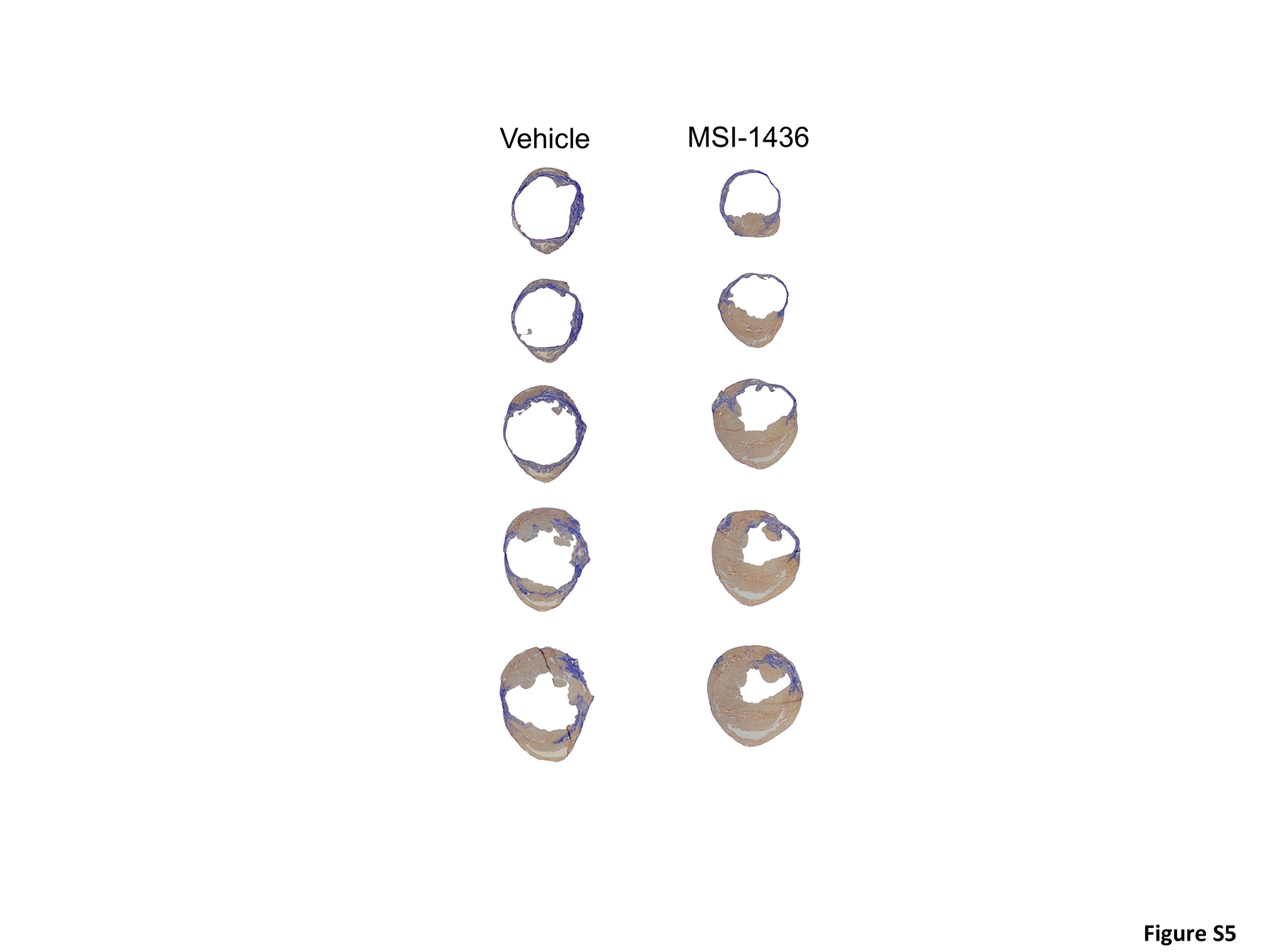

Supplement: Supplementary file 6 — Supplementary Figure S5 [file 41536_2017_8_MOESM6_ESM.jpg]

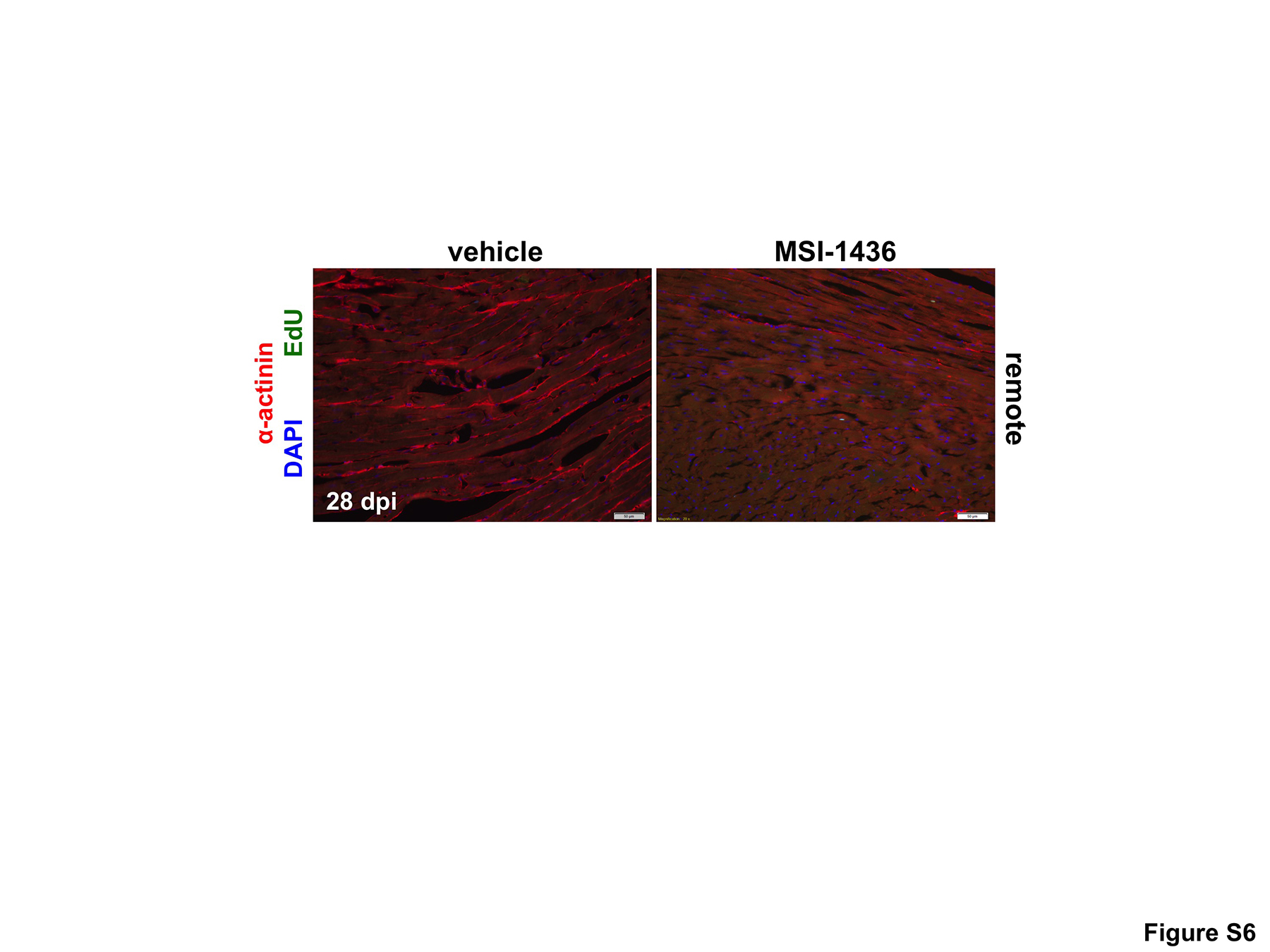

Supplement: Supplementary file 7 — Supplementary Figure S6 [file 41536_2017_8_MOESM7_ESM.jpg]
